# Online Hemodiafiltration Compared to Conventional Hemodialysis in Critically Ill Patients

**DOI:** 10.1016/j.ekir.2022.08.007

**Published:** 2022-08-24

**Authors:** Julie Piotte, Félix Louis, Dimitry Buyansky, Eric Mereniuk, Renée Lévesque, Ron Wald, Jean-François Cailhier, Jean-Maxime Côté, William Beaubien-Souligny

**Affiliations:** 1 Department of Medicine, Université de Montréal, Montréal, Canada; 2 Department of Medicine, Université de Sherbrooke, Montréal, Canada; 3 Division of Nephrology, Centre Hospitalier de l’Université de Montréal, Montréal, Canada; 4 Division of Nephrology, St. Michael’s Hospital, Toronto, Canada; 5 Centre de recherche du Centre Hospitalier de l’Université de Montréal, Montréal, Canada

**Keywords:** acute kidney injury, convective clearance, hemodiafiltration, hemodialysis, inflammation, renal replacement therapy

## Abstract

**Introduction:**

Online hemodiafiltration (HDF) has been increasingly used for improved clearance of middle molecular weight toxins. The impact of this mode of clearance is unknown in critically ill patients. We aimed to determine whether the use of HDF in acute kidney injury (AKI) is associated with lower mortality and improved kidney recovery up to 90 days after initiation of therapy.

**Methods:**

Single-center retrospective cohort study using data from 2017 to 2020 of adults with AKI who initiated intermittent renal replacement therapy (IRRT) in the intensive care unit (ICU), using either hemodialysis (HD) or HDF depending on the maintenance status of the water system without regards for patient characteristics. We assessed association with patient-events and session-events using time-dependent Cox models and general estimating equations models, respectively.

**Results:**

We included 182 adults with AKI for whom 848 IRRT sessions were performed in the ICU. The 90-day mortality rate was 43 of 182 (24.6%). There was no significant association with the use of HDF and mortality (adjusted hazard ratio [aHR]: 0.85 (0.43; 1.67) *P* = 0.64), kidney recovery (aHR: 1.18 (0.76; 1.84) *P* = 0.47), or intradialytic hypotension (adjusted odds ratio [aOR]: 0.91 confidence interval [CI]: 0.64–1.28 *P* = 0.58). HDF treatment was associated with a lower rate of subsequent vasopressor use (aOR: 0.60 CI: 0.36–0.99 *P* = 0.047) and a greater reduction of the neutrophil-to-lymphocyte ratio (NLR) following the first session (−15.0% vs. +5.1%, *P* = 0.047) but was also associated with increased risk of filter thrombosis during treatment (aOR: 2.42 CI: 1.67–3.50 *P* < 0.001).

**Conclusion:**

The use of HDF in the setting of AKI was not associated with a differential risk of mortality or kidney recovery.

## Graphical abstract

**Figure:**
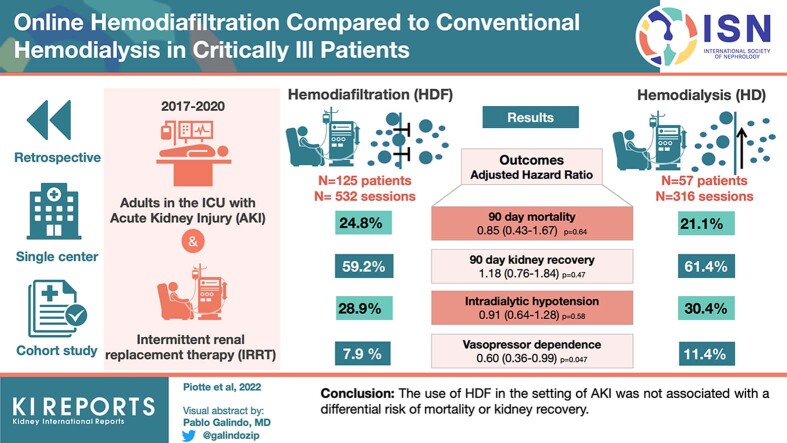

Severe AKI in critically ill patients requiring renal replacement therapy (RRT) has a poor prognosis,^1^^,^^2^ with short-term mortality up to 60% when there is multiple organ dysfunction.^3^, ^4^, ^5^, ^6^ Survivors may develop kidney scarring, including tubular atrophy and interstitial fibrosis, which can lead to chronic kidney disease.^7^ Despite advances in RRT technology, only slight improvements in patient outcomes have been reported and mortality rate remains high.^8^

Whereas continuous RRT is generally preferred in patients with hemodynamic instability,^3^^,^^9^ IRRT is generally deployed in survivors after stabilization. Conventional HD provides clearance of small solutes mainly via a diffusion process but has limited efficiency in removing larger molecules like cytokines.^10^ Online HDF combines diffusive and convective solute removal in a single treatment but requires the production of ultrapure substitution fluid.^11^ In the setting of end-stage kidney disease (ESKD), the use of HDF tends to reduce inflammatory activity and improve hemodynamic tolerance to IRRT.^12^ Immune modulation has been explored in the setting of AKI because inflammation plays a prominent role in the initial pathophysiology of AKI and may also contribute to the maintenance of the renal insult^13^ and extrarenal organ dysfunction.^14^ Therefore, HDF appears a promising modality for inflammatory modulation, although it may also be associated with unforeseen risks. Because the use of convective clearance as a possible mode of clearance in IRRT is relatively recent, there is a paucity of data comparing the outcomes between HDF and HD in the context of AKI. A recent meta-analysis emphasized the need for further studies evaluating the impact of convective IRRT modalities in the ICU.^15^

Maintenance of the water system may temporarily preclude the use of HDF and force the use of HD, thereby creating a context mimicking random allocation corresponding to a natural experiment. In this study, we aimed to determine whether the use of online HDF in critically ill patients with AKI is associated with improved outcomes as compared to HD. We first investigated whether HDF exposure was associated with lower short-term mortality. We also evaluated whether HDF was associated with kidney recovery, RRT complications and overall inflammatory burden.

## Methods

### Setting

The research is a single-center retrospective cohort study which took place at the Centre Hospitalier de l’Université de Montréal, a large academic centre, using data from January 2017 to August 2020.

### Population

The study included any patient who initiated IRRT in the ICU or cardiac ICU in the context of AKI from January 2017 to August 2020 at the Centre Hospitalier de l’Université de Montréal. AKI was defined by the Kidney Disease: Improving Global Outcome criteria as an increase in serum creatinine by 27 μmol/l or more within 48 hours or an increase in serum creatinine to 1.5 times baseline or more within the last 7 days, or documented urine output less than 0.5 ml/kg/h for 6 hours.^16^ Patients were excluded if they had IRRT for AKI in the previous 90 days, if they were recipients of RRT for ESKD, or if RRT was not initiated in the ICU.

Patients received either conventional HD or HDF depending on the maintenance status of the water purification on the day of initiation and subsequent dialysis. The maintenance status of the water system was the only limiting factor to receive HD instead of HDF and patient characteristics had no regards to the choice of therapy initiated. At our institution, the temporary exclusive use of HD is enforced when microbiologic requirements for ultrapure water (International Organization for Standardization)^17^ are not met, or when maintenance procedures susceptible to affect the quality of the water are performed. All IRRT treatments were delivered with the 5008 High-Volume HDF machine from Fresenius Medical Care, using either the High-Flux F×1000 dialyzer (Effective surface area: 2.2 m^2^, Fresenius Medical Care; Germany) or the Evodial 2.2 dialyzer (Effective surface area: 2.15 m^2^, Baxter, Canada). Substitution fluid rate during treatment is optimized using the AutoSub program (Fresenius Medical Care; Germany). The substitution fluid can be administered before (predilution) or after (postdilution) the filter at the discretion of the physician. Dalteparin is used as the default anticoagulant when no contraindication is present per the clinical judgment of the attending clinician. As opposed to conventional HD or predilution HDF, postdilution HDF is rarely performed without anticoagulation.

### Data Collection

Dialysis-specific electronic medical records (NephroInsight; Constellation Kidney Group) were used to collect data about detailed parameters of each acute IRRT session performed, including the clearance mode, substitution volume, ultrafiltration, mean blood flow rate, use of dalteparin, and total duration of the session. The patients’ charts were reviewed to collect information regarding demographic variables, baseline health status, including the Charlson comobidity index,^18^ and information about the severity of illness at the time of IRRT initiation, including the prior use of continuous RRT and the Sequential Organ Failure Assessment score.^19^ Medical notes were reviewed to document the presence of active sepsis or cardiogenic shock based on the list of diagnosis of the attending physician. Baseline kidney function was assessed by estimated glomerular filtration rate calculated by the chronic kidney disease-epidemiology collaboration formula^20^ from the most recent outpatient serum creatinine measurement prior to hospital admission. If no creatinine was available for the past year, the first value after admission was used. Before each IRRT treatment performed in the ICU, we collected information about baseline arterial blood pressure, vasopressor support, and use of mechanical ventilation, as well as the following laboratory parameters: lymphocyte count, neutrophil count, and monocyte count. Vasopressor support was quantified using the vasoactive-inotropic score defined as [vasoactive-inotropic score = dopamine dose + dobutamine dose + 100 × epinephrine dose + 10 × milrinone dose + 10,000 × vasopressin dose + 100 × norepinephrine dose] where all doses are in μg/kg/min except vasopressin dose which is in U/kg/min.^21^, ^22^, ^23^, ^24^ For each IRRT treatment in the ICU, we collected information about the minimal arterial blood pressure and maximal vasoactive support reached during the treatment. After ICU discharge, basic information about IRRT sessions performed up to 90 days were collected, including dialysis modality and substitution volume. We collected information about mortality (date) and receipt of RRT until 90 days after initiation of IRRT.

### Exposures, Outcomes and Definitions

The primary exposure was the receipt of HDF either via predilution or postdilution method. Corrected convection volume was calculated in liters by adding the substitution volume to the net ultrafiltration volume removed during the session. When predilution method was used, the substitution volume was divided by two to account for the reduced clearance associated with this method in accordance with usual practice in patients with ESKD.^25^, ^26^, ^27^

The primary outcome was all-cause mortality up to 90 days. Secondary outcomes included kidney recovery up to 90 days, defined as the discontinuation of RRT for a ≥14-day period with the date of recovery defined as the day of the last RRT treatment. Intradialytic hypotension was defined as systolic blood pressure of less than 90 mmHg during IRRT, and/or initiation/increase in vasopressor support. This definition has been previously associated with in-hospital mortality.^28^ Subsequent vasopressor requirement was defined as the presence of vasopressor support immediately before the initiation of the subsequent IRRT session in the ICU. Filter clotting was defined as stage 3 (partially coagulated) and stage 4 (completely coagulated) as routinely noted by the dialysis specialized nursing personnel. The NLR was calculated as the ratio of the absolute neutrophil count to the lymphocyte count measured the morning before IRRT treatments in the ICU. Finally, ICU-free days were calculated by identifying all days during which patients were alive and out of the ICU.

### Statistical Analysis

In order to determine if the use of HDF is associated with mortality up to 90 days, a Cox proportional hazard model for counting processes with HDF exposure as a time-dependant variable was used. Consequently, the exposure to HDF as a variable was updated at each time interval (day) based of the last use of RRT, and the resulting HR represents the instantaneous risk of death during the following time period and are presented with 95% confidence intervals. Univariable analysis and multivariable analysis were then performed. Adjustment variables were prespecified in the protocol and included age, sex, Charlson index, baseline estimated glomerular filtration rate, prior use of continuous RRT, Sequential Organ Failure Assessment score^19^ at IRRT initiation, admission diagnostic category (medical or surgical), and the presence of sepsis and/or heart failure during ICU stay. The same approach was used to determine if exposure to HDF was associated with improved rate of kidney recovery up to 90 days. For this analysis, patients who died were censored at the time of death. Patients who were transferred to another center before 90 days without subsequent contact were considered lost to follow-up and censored at that time for the purpose of the analysis. Survival and kidney recovery were also plotted using Kaplan-Meier curves based on the initial IRRT modality used in the ICU compared with the log-rank test.

To determine if HDF was associated with a lower incidence of intradialytic hypotension, filter clotting, or persistent vasopressor requirement compared with HD, we used generalized estimating equation analysis with a logistic link function with an unstructured correlation matrix (up to 100 iterations). This type of analysis accounts for the repeated measures design, implying that the sample was not independent (multiple IRRT sessions per patient). For the association with the risk of intradialytic hypotension, adjustments for vasoactive-inotropic score immediately before the start of the IRRT session, the time since initial IRRT initiation in days, and the use of mechanical ventilation was performed. For the association with the risk of filter clotting, adjustment for dalteparin use, platelet count, session duration, and mean blood flow was performed. For the association with persistent vasopressor support, adjustment was performed with the time in days since IRRT initiation and vasoactive-inotropic score immediately at the initial IRRT initiation (before first session). In order to determine if the use of HDF compared to HD was associated with the NLR, we compared the relative change in NLR in percent from the morning before the initiation of IRRT to immediately before the next IRRT session between HDF and HD as the initial modality using the Mann-Withney U test for independent samples. Finally, ICU-free days were compared between HDF and HD as the initial modality using Mann-Witney U independent sample test.

As a sensitivity analysis, all regression analyses were repeated using corrected convection volume in liters, using half of the convective volume for the HDF predilution method, as a continuous variable and the exposure variable. This was done because the potential beneficial effect of HDF is related to the total convective clearance achieved. We also performed Cox regression adjusted for the same covariables included in the primary analysis comparing initial IRRT modality for 90-day mortality and kidney recovery in the subgroup of patients with sepsis at IRRT initiation.

Descriptive data are presented in number (%) for dichotomous variables and in mean ± SD or median and interquartile range for continuous variables, where appropriate. Simple comparisons between groups were assessed using Pearson Chi-square or Fisher exact test (when minimal observation per cell was <5) for dichotomous variables, or independent-sample *t* test or Mann-Whitney U test for continuous variables. The normality of distribution for continuous variables was assessed using Q-Q plots. Analyses were performed in SPSS 27(IBM; Armonk, NY) and SAS 9.4 (SAS Institute, Cary, NC).

## Results

### Patients Characteristics

**Figure 1:**
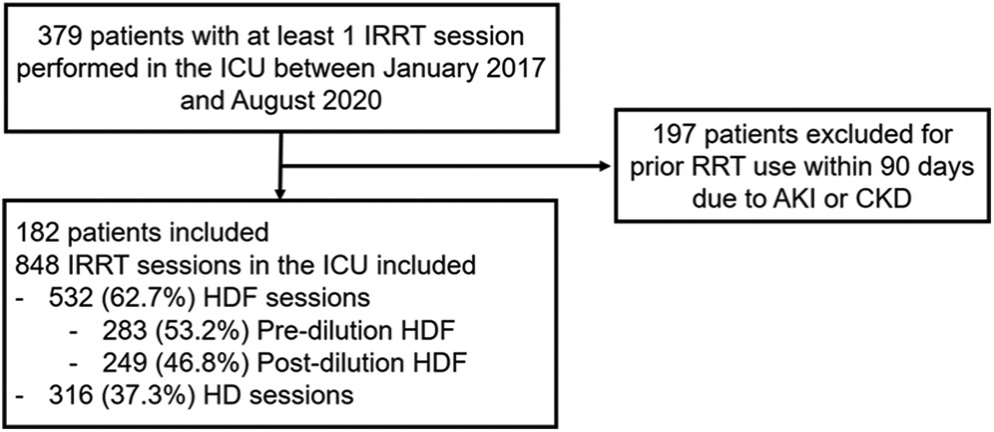
Patient inclusion flowchart. AKI, acute kidney injury; CKD, chronic kidney disease; HD, hemodialysis; HDF, hemodiafiltration; ICU, intensive care unit; IRRT, intermittent renal replacement therapy; RRT, renal replacement therapy.

Between January 2017 and August 2020, 379 patients who had RRT in the ICU were screened. Of these patients, 197 (52%) were not eligible for inclusion because IRRT was performed before ICU admission, either for AKI or ESKD. Therefore, we included 182 adults with AKI for whom a total of 848 IRRT sessions were performed in the ICU (Figure 1). Among these patients, 57 (31.3%) initiated HD, whereas 125 (68.7%) commenced HDF. As shown in Table 1, the majority were men 126 (69%), the median age of 65 [56–73] years, and a baseline median eGFR of 61 [30–90] ml/min per 1.73 m^2^. Baseline characteristics of both groups were similar in terms of comorbidities (Table 1) apart from a history of previous stroke or transient ischemic attack, which was more important in the group initiated on HD (9 of 57 [15.8%] vs. 7 of 125 [5.6%], *P* = 0.024). The severity of illness, the primary diagnosis of admission, the initial RRT choice between continuous or intermittent modality, the use of mechanical ventilation or vasopressors, and the Sequential Organ Failure Assessment score at IRRT initiation were all similar. The duration of follow-up was also similar (median: 90 [90; 90] days in both groups, *P* = 0.20).

**Table 1:** Patients characteristics in relationship with the initial IRRT used in the intensive care unit

Characteristics		All patients (*N* = 182)	Initiated on HD (*N* = 57)	Initiated on HDF (*N* = 125)	*P*-value
Female		56 (30.8%)	21 (36.8%)	35 (28.0%)	0.23
Age (yr)		65 [56; 73]	65 [55; 72]	65 [57; 73]	0.49
Body mass index (kg/m^2^)		28.1 [23.8; 33.0]	27.9 [23; 34]	28.1 [24.3; 33.0]	0.76
Comorbidities					
Hypertension		116 (63.7%)	41 (71.9%)	75 (60.0%)	0.14
Diabetes	Without end organ damage	33 (18.1%)	9 (15.8%)	24 (19.2%)	0.48
	With end-organ damage	49 (26.9%)	15 (26.3%)	34 (27.2%)	
Coronary artery disease		68 (37.4%)	18 (31.6%)	50 (40%)	0.20
Congestive heart failure		41 (22.5%)	10 (17.5%)	31 (24.8%)	0.34
Chronic pulmonary obstructive disease		31 (17.0%)	10 (17.5%)	21 (16.8%)	0.74
Previous stroke/transient ischemic attack		16 (8.8%)	9 (15.8%)	7 (5.6%)	0.024
Liver disease	Mild	11 (6.0%)	5 (8.8%)	5 (4.8%)	0.69
	Moderate-to-severe	25 (13.7%)	8 (14.0%)	17 (13.6%)	
Cancer	Localized	18 (9.9%)	7 (12.28%)	11 (8.8%)	0.56
	Metastatic	10 (5.5%)	2 (3.51%)	8 (6.4%)	
Baseline estimated glomerular filtration rate (ml/min/1.73 m^2^)		61 [30; 90]	61 [33; 89]	60 [30; 93]	0.73
Charlson score (points)		4.1 (2.5)	4.1 (2.7)	4.2 (2.4)	0.80
Current hospitalization–severity of illness					
Category of primary diagnosis	Medical	132 (72.5%)	42 (73.7%)	90 (72.0%)	0.81
	Surgical	50 (27.5%)	15 (26.3%)	35 (28.0%)	
Initial RRT modality in the ICU	IRRT	78 (42.9%)	27 (47.4%)	51 (40.8%)	0.41
	Continuous RRT	104 (57.1%)	30 (52.6%)	74 (59.2%)	
	Duration of CRRT before transition (d)	7 [5; 13]	8 [4; 13]	7 [5; 13]	
Mechanical ventilation at IRRT initiation		76 (41.8%)	20 (35.1%)	56 (44.8%)	0.47
Vasopressor support at IRRT initiation		50 (27.5%)	14 (24.6%)	36 (28.8%)	0.41
Total SOFA score at IRRT initiation (points)		4.9 (3.7)	4.5 (3.4)	5.0 (3.8)	0.52
Characteristics of IRRT session received in the ICU					
No. of sessions in the ICU per patient		3 [1; 5]	3 [1; 6]	3 [2; 5]	0.87
% HDF in the ICU		92% [33; 100%]	0% [0; 39%]	100% [88; 100%]	<0.0001
Initial dilution method	Predilution			77 (61.6%)	
	Postdilution			48 (38.4%)	
Median corrected convection volume per treatment (L)		15.6 [6.8; 23.2]	2.9 [1.5; 10.7]	19.0 [14.1; 25.3]	<0.0001
Rate of intradialytic hypotension		20.0% [0.0%; 50%]	18.4% [0.0%; 66.7%]	20.0% [0.0%; 50.0%]	0.94
Rate of partial or complete filter thrombosis		22.0% [0.0%; 50.0%]	0.0% [0.0%; 25.0%]	40.0% [0.0; 66.7%]	<0.0001
Clinical outcomes					
Death within 90 d		43 (23.6%)	12 (21.1%)	31 (24.8%)	0.67
Kidney recovery within 90 d in survivors		109/166 (65.7%)	35/53 (66.0%)	74/113 (65.5%)	0.85
Median duration of follow-up (d)		90 (90; 90)	90 (90; 90)	90 (90; 90)	0.20

CRRT, continuous renal replacement therapy; HD, hemodialysis; HDF, hemodiafiltration; ICU, intensive care unit; IRRT, intermittent renal replacement therapy; RRT, renal replacement therapy; SOFA, Sequential Organ Failure Assessment.

Data is presented in *N* (%), median [interquartile range] or mean (SD).

### Sessions Characteristics

Among the IRRT sessions performed in the ICU (*N* = 848), 316 (37.3%) were HD and 532 (62.7%) were HDF, including 283 (53.2%) performed in predilution mode and 249 (46.8%) performed in postdilution mode. Session duration, dalteparin use, and mean blood flow rate were similar between HD and HDF sessions (Supplementary Table S1). The median corrected convection volume per session for HDF was 22.7 L (17.7–27.5) compared to 2.1 L (0.9–3.1) in HD session (*P* < 0.001).

### Mortality at 90 Days

**Figure 2:**
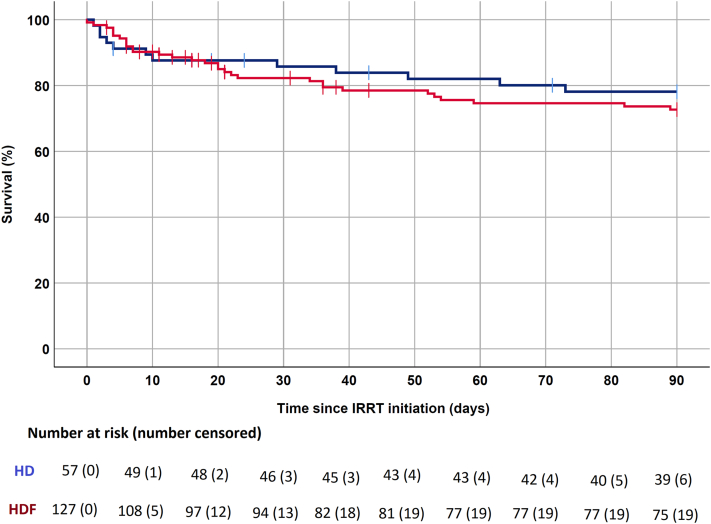
Survival up to 90 days after initiation of IRRT according to initial modality. Red: online hemodiafiltration, Blue: conventional hemodialysis, Log-rank: *P* = 0.49. HD, hemodialysis; HDF, hemodiafiltration; IRRT, intermittent renal replacement therapy.

The overall mortality rate was 43 of 182 (23.6%), including 12 (21.1%) who received HD initially compared to 31 (24.8%) patients among those who received HDF initially. There was no difference in the risk of death according to the initial IRRT modality used as shown in Figure 2 (Log-rank *P* = 0.49). Results were similar when only patients who received HD or HDF exclusively (*n* = 122) in the ICU were analyzed (*P* = 0.57).

When HDF exposure was considered as a time-varying covariate, no significant association with mortality in the first 90 days was found before or after adjustment for baseline characteristics and severity of illness (aHR: 0.85 [95% CI, 0.43; 1.67] *P* = 0.64) (Table 2). Similarly, there was no association between corrected convection volume and mortality (aHR: 0.89 per 5 liter increments [95% CI, 0.78; 1.03] *P* = 0.11) (Supplementary Table S2).

**Table 2:** Association between IRRT modality as a time-varying exposure and mortality within 90 days

Variable	Univariable (HR, [95% CI])	*P*-value	Multivariable (aHR, [95% CI])	*P*-value
Conventional hemodialysisOnline hemodiafiltration	Ref 0.93 (0.48; 1.78)	0.82	Ref 0.85 (0.43; 1.67)	0.64
Age	1.03 (1.00–1.05)	0.050	1.04 (1.00; 1.08)	0.06
Female sex	1.19 (0.67; 2.11)	0.56	2.28 (1.08; 4.84)	0.032
Charlson comorbitiy score	1.13 (1.00–1.27)	0.052	1.11 (0.93; 1.33)	0.24
Baseline eGFR	1.00 (0.99; 1.01)	0.76	1.01 (1.00; 1.02)	0.045
Initially started on CRRT	0.83 (0.50; 1.38)	0.47	0.53 (0.26; 1.09)	0.08
SOFA score at IRRT initiation	1.08 (1.00–1.16)	0.051	1.14 (1.04; 1.25)	0.007
Admission diagnostic		0.22		0.06
Surgical	Ref		Ref	
Medical	1.62 (0.75; 3.49)		2.35 (0.984; 5.636)	
Associated clinical conditions				
Sepsis	1.40 (0.80; 2.45)	0.25	1.11 (0.54; 2.29)	0.78
Heart failure	0.57 (0.25; 1.32)	0.19	0.33 (0.09; 1.17)	0.09
Both	0.70 (0.21; 2.29)	0.55	0.23 (0.03; 1.83)	0.16

aHR, adjusted hazard ratio; CI, confidence interval; CRRT, continuous renal replacement; eGFR, estimated glomerular filtration rate; HR, hazard ratio; IRRT, intermittent renal replacement therapy; SOFA, Sequential Organ Failure Assessment.

Cox regression model for count processes with HDF exposure as a time-varying covariate. One patient was excluded from the analysis for loss to follow-up after a single IRRT session (*N* = 181).

In a sensitivity analysis, there was no difference in the risk of death according to the initial IRRT modality in the subgroup of patients who had sepsis (*n* = 62) at IRRT initiation (aHR: 2.08 [0.66–6.57] *P* = 0.21).

### Kidney Recovery at 90 Days

**Figure 3:**
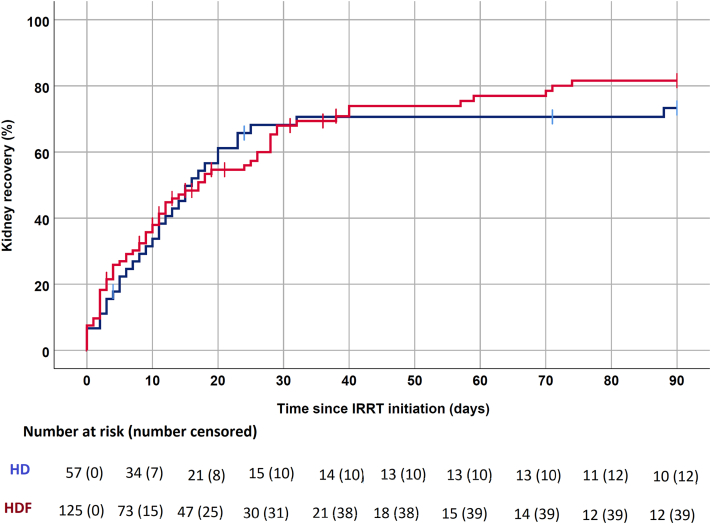
Kidney recovery up to 90 days after initiation of IRRT according to initial modality. Red = online hemodiafiltration, Blue: conventional hemodialysis, Log-rank: *P* = 0.61). HD, hemodialysis; HDF, hemodiafiltration; IRRT, intermittent renal replacement therapy.

Overall, 109 of 182 (59.9%) patients had kidney recovery at 90 days follow-up, including 35 of 57 (61.4%) patients with HD as their initial modality, and 74 of 125 (59.2%) for HDF. There was no difference in the rate of kidney recovery according to the initial IRRT modality used as shown in Figure 3 (Log-rank *P* = 0.61). Results were similar when only patients who received HD or HDF exclusively in the ICU (*n* = 122) were analyzed (*P* = 0.49).

HDF as a time-varying exposure was not associated with improved 90-day kidney recovery (aHR: 1.18 [95% CI, 0.76; 1.84] *P* = 0.47) (Table 3). Similarly, there was no association with corrected convection volume and kidney recovery (aHR: 0.89 per 5L increments [0.78; 1.03] *P* = 0.11) (Supplementary Table S3).

**Table 3:** Association between IRRT modality as a time-varying exposure and kidney recovery until 90 days

Variable	Univariable (HR, [95% CI])	*P*-value	Multivariable (aHR, [95% CI])	*P*-value
Conventional hemodialysis	Ref		Ref	
Online hemodiafiltration	1.19 (0.77; 1.84)	0.44	1.18 (0.76; 1.84)	0.47
Age	0.99 (0.97; 0.999)	0.033	0.99 (0.97; 1.01)	0.51
Female sex	1.07 (0.71; 1.61)	0.75	1.32 (0.84; 2.08)	0.23
Charlson comorbidity score	0.95 (0.87; 1.02)	0.16	1.02 (0.92; 1.15)	0.68
Baseline eGFR	1.01 (1.00; 1.01)	0.007	1.01 (1.00; 1.02)	0.024
Initially started on CRRT	1.00 (0.83; 1.22)	0.98	0.66 (0.43; 1.02)	0.06
SOFA score at IRRT initiation	1.01 (0.96; 1.06)	0.70	1.01 (0.95; 1.07)	0.052
Admission diagnostic				
Surgical	Ref		Ref	
Medical	1.06 (0.86; 1.32)	0.57	1.27 (0.78; 2.06)	0.34
Context at ICU admission				
Sepsis	1.23 (0.78; 1.93)	0.38	1.36 (0.84; 2.20)	0.21
Heart failure	0.93 (0.56; 1.55)	0.78	1.03 (0.60; 1.79)	0.91
Both	1.69 (0.83; 3.46)	0.15	1.98 (0.94; 4.18)	0.07

aHR, adjusted hazard ratio; CI, confidence interval; CRRT, continuous renal replacement; eGFR, estimated glomerular filtration rate; HR, hazard ratio; IRRT, intermittent renal replacement therapy; SOFA, Sequential Organ Failure Assessment.

Cox regression model for count processes with HDF exposure as a time-varying covariate. Two patients were excluded from the analysis for kidney recovery and loss to follow-up after a single IRRT session (*N* = 180).

In a sensitivity analysis, there was no difference in the kidney recovery according to the initial IRRT modality in the subgroup of patients who had sepsis (*n* = 62) at IRRT initiation (aHR: 0.75 [CI: 0.33; 1.71] *P* = 0.50).

### Intradialytic Hypotension and Vasopressor Use

Intradialytic hypotension occurred in 250 of 848 sessions (29.5%), including 96 of 316 HD sessions (30.4%) and 154 out of 532 HDF sessions (28.9%). When controlling for possible confounding variables, such as duration of IRRT, vasopressor-inotropic score at the start of the session, and the presence of mechanical ventilation, there was no significant association between HDF and the occurrence of intradialytic hypotension (aOR: 0.91 [CI: 0.64–1.28] *P* = 0.58) (Table 4). Similar results were obtained in regard to corrected convection volume received as well as the dilution method (Supplementary Table S4 and Table S5).

**Table 4:** Association between IRRT modality and intradialytic hypotension in the ICU

Variable	Univariable (OR, [95% CI])	*P*-value	Multivariable (aOR, [95% CI])	*P*-value
Conventional hemodialysis	Ref		Ref	
Online hemodiafiltration	0.93 (0.66–1.33)		0.91 (0.64–1.28)	0.58
Use of mechanical ventilation	1.38 (0.91–2.08)	0.13	1.28 (0.85–1.93)	0.23
Vasopressor-inotropic score before IRRT initation (per 1 point increase)	1.07 (1.00–1.14)	0.041	1.06 (0.89–1.13)	0.06
Duration since IRRT initiation (d)	0.99 (0.97–1.01)	0.25	0.99 (0.97–1.01)	0.30

aOR, adjusted odds ratio; CI, confidence interval; IRRT, intermittent renal replacement therapy; OR, odds ratio.

Data from 848 IRRT sessions in 182 patients was included.

Subsequent or persistent dependence on vasopressor was observed following 78 of 848 sessions (9.2%), including 36 of 316 (11.4%) following an HD session and 42 of 532 (7.9%) following an HDF session. HDF was associated with a lower rate of subsequent dependence on vasopressor (aOR: 0.60 [95% CI, 0.36–0.99] *P* = 0.047) (Table 5)*.* In sensitivity analyses, there was no association between subsequent vasopressor dependence and either dilution method or corrected convection volume (Supplementary Table S6 and S7).

**Table 5:** Association between IRRT modality and subsequent vasopressor dependence in the ICU

Variable	Univariable (OR, [95% CI])	*P*-value	Multivariable (aOR, [95% CI])	*P*-value
Conventional hemodialysis	Ref		Ref	
Online hemodiafiltration	0.61 (0.37–0.98)		0.600 (0.36–0.99)	0.047
Vasopressor-inotropic score before IRRT initation (per 1 point increase)	1.03 (0.99–1.07)	0.13	1.028 (0.99–1.06)	0.12
Duration since IRRT initiation (d)	0.98 (0.90–1.06)	0.59	0.967 (0.89–1.05)	0.43

aOR, adjusted odds ratio; CI, confidence interval; IRRT, intermittent renal replacement therapy; OR, odds ratio.

Data from 848 IRRT sessions in 182 patients was included.

### Filter Clotting

Partial or complete filter thrombosis occurred in 259 of 848 sessions (30.5%), including 62 of 316 (19.6%) for HD and 197 of 532 (37.0%) for patients on HDF. Filter thrombosis occurred more often with predilution HDF than with postdilution HDF (141/283 [49.8%] vs. 56/250 [22.4%] *P* < 0.001). The occurrence of filter thrombosis was more frequent with predilution HDF compared to HD without dalteparin administration (141/283 [49.8%] vs. 46/192 [24.0%] *P* < 0.001), as well with postdilution HDF compared to HD with dalteparin administration (56/250 [22.4%] vs. 16/124 [12.9%] *P* = 0.03). After adjustment for dalteparin use, mean blood flow, platelet count, and session duration, there was a significant difference in the occurrence of these events between both groups (aOR 2.42 [95% CI, 1.67–3.50] *P* ≤ 0.001) (Table 6). Nevertheless, in a sensitivity analysis, we observed that there was no association between filter clotting and duration of treatment (β = −3.5 CI: −12.7; 5.5 *P* = 0.44).

**Table 6:** Association between IRRT modality and filter thrombosis during treatment

Variable	Univariable (OR, [95% CI])	*P*-value	Multivariable (aOR, [95% CI])	*P*-value
Conventional hemodialysis	Ref		Ref	
Online hemodiafiltration	2.11 (1.48–3.00)		2.42 (1.67-3.50)	<0.001
Dalteparin use (yes/no)	0.65 (0.44–0.97)	0.03	0.64 (0.44-0.93)	0.02
Platelet level (per 10 increments)	1.01 (0.909–1.13)	0.83	1.01 (0.99-1.02)	0.32
Session duration (per 1h increments)	1.09 (0.83–1.43)	0.54	1.01 (0.82-1.23)	0.94
Mean blood flow (per 100 ml/min increments)	0.95 (0.77–1.17)	0.61	0.87 (0.65-1.16)	0.34

aOR, adjusted odds ratio; CI, confidence interval; IRRT, intermittent renal replacement therapy; OR, odds ratio.

Data from 848 IRRT sessions in 182 patients was included.

### Neutrophil-to-Lymphocyte Ratio

There was no significant difference between the absolute NLR *per se* after the first IRRT session (9.2 [4.6; 16.0] vs. 11.0 [5.8; 17.2] *P* = 0.29) (Table 7). Nevertheless, we observed a significant change in the NLR following the first IRRT session in those who received HDF compared to HD (−14.2% [−48.5; 12.1%] vs. +5.2% [−38.1; 69.1%] *P* = 0.048).

**Table 7:** Neutrophil-to-lymphocyte before and after the first IRRT session performed in the ICU

Variable	All	HD	HDF	*P*-value
NLR before IRRT initation	11.2 [7.1; 20.7]	10.6 [6.7; 20.1]	11.3 [7.1; 20.8]	0.43
NLR before the following IRRT session	9.8 [5.1; 16.3]	11.0 [5.8; 17.2]	9.2 [4.6; 16.0]	0.29
Relative change in NLR	−9.7% [−43.6; 31.7%]	+5.2% [−38.1; 68.1%]	−14.2% [−48.5; 12.1%]	0.048

HD, hemodialysis; HDF, hemodiafiltration; ICU, intensive care unit; IRRT, intermittent renal replacement therapy; NLR, neutrophil-to-lymphocyte ratio.

*N* = 663 (Only IRRT session performed in the ICU with data from a subsequent session were included.

### ICU-free Days

There was no significant difference in ICU-free days through day 90 between patients who initiated HDF compared to patients who first initiated HD (median: 79 [61; 86] days vs. 78 [46; 85] days, *P* = 0.59).

## Discussion

In this study, we compared HDF to HD performed in the ICU in patients with AKI. We found that 90-day mortality and kidney recovery were not associated with HDF exposure. Furthermore, we did not observe less intradialytic hypotension, although HDF was associated with a reduction in vasopressor use. The use of HDF was associated with greater improvement of NLR following the first IRRT session, and a higher rate of filter thrombosis.

High-volume hemofiltration has been proposed as a method to provide clearance of proinflammatory cytokines and other noxious endogenous products that may mediate adverse outcomes in critically ill patients.^29^^,^^30^ Trials investigating the use of continuous hemofiltration have not shown significant differences in patient outcomes.^31^^,^^32^ In contrast, the possibility to perform high volumes of convective clearance using IRRT is more recent with the development of online HDF. In a recent meta-analysis comparing conventional HD to intermittent convective therapies,^15^ we reported that only 3 small scale exploratory trials ever compared HD to HDF.^33^, ^34^, ^35^ Most interestingly, pooling the results of these trials revealed a higher risk of in-hospital death with the use of HDF (Risk ratio: 2.07 CI: 1.04–4.11).^15^ Therefore, further investigation was urgently required to determine the safety and potential benefits of HDF in an ICU setting. In this cohort, we did not observe an increased risk of death with the use of HDF. Nevertheless, we observed a trend toward increased mortality risk in patients with sepsis who first initiated HDF that should be further investigated.

In HDF, the clearance of solutes is highly dependent on the blood flow rate and the total convective volume achieved during dialysis. Previous studies have shown that postdilution HDF with high convection volumes (>22 l/session) has survival benefits in maintenance dialysis recipients.^11^^,^^36^ These benefits, however, were not reproduced in patients who received lower convection volumes.^11^^,^^37^ In the present cohort, we observed a median convection volume of 22.7 liters per ession, suggesting that about half of HDF sessions failed to achieve the targeted volume. Indeed, a high convection volume might be difficult to achieve in the context of the ICU. Recent studies associated failure to reach >22 liters per session convection volumes with the use of central venous catheters, shorter treatments, and lower blood flow rate.^38^^,^^39^ Because IRRT is conducted with central catheters in the setting of AKI, the ability to optimize convection volume without prolonging treatment may be limited.

Though online HDF has been shown to reduce the occurrence of intradialytic hypotension in patients with chronic ESKD,^40^ we did not observe a lower rate during HDF compared to HD although a reduction of subsequent vasopressor use was documented. Though some trials suggested improved hemodynamic parameters, including reduced vasopressor requirements with ultrafiltration,^29^^,^^30^^,^^41^ it is unclear if this is mediated through convective clearance itself or through other mechanisms such as the thermal effect of infusing large quantities of replacement fluid.^42^ The fact that HDF was associated with reduced subsequent need for vasopressor in our study supports raising the hypothesis that the beneficial hemodynamic effect in this setting is delayed. Whether this is related to an improvement in inflammatory status could be further studied. Furthermore, the clinical effect remains unclear because no difference in ICU-free days was demonstrated.

NLR is a marker of inflammation and is associated with adverse outcomes in AKI patients.^43^^,^^44^ The greater reduction in NLR following the first HDF session compared with HD might indicate a favorable immunomodulatory effect of high-volume convective clearance. HDF allows for clearance of proinflammatory molecules such as cytokines^34^ although it is unclear if this affects the overall inflammatory status in critically ill patients.^29^^,^^30^ Several trials comparing low-flux HD, high-flux HD and HDF have shown a reduction of the number of CD14+ CD16+ monocytes and their proinflammatory factors in HDF cohorts.^45^, ^46^, ^47^ When adjusted to achieve high convective volumes, HDF tends to improve inflammatory status when compared to HD in maintenance dialysis.^15^

We observed frequent filter coagulation, which occurred in approximating 30% of sessions in the ICU. This surprising result might be due to our broad definition of filter coagulation which included partially coagulated filters. When accounting for dalteparin use, HDF exposure was still associated with an increased risk of filter coagulation. Hemofiltration during HDF results in a higher hematocrit in the later portion of the filter, which may promote thrombosis. Furthermore, patients receiving IRRT in the ICU are generally more inflammatory and at higher risk of bleeding. They are therefore more prone to have an hypercoagulable state but also less likely to receive optimal circuit anticoagulation. Higher dosage of low molecular weight heparin are necessary to achieve effective anticoagulation in postdilution HDF compared to HD.^48^ Therefore, the higher filter thrombosis rate of postdilution HDF may be partially amendable through careful optimization of anticoagulation when no contraindication is present. We also observed a higher rate of filter thrombosis with the use of predilution HDF compared to HD performed without anticoagulation, a finding that was previously reported.^49^ Convective techniques have been demonstrated to result in greater levels of D-dimers and thrombin-antithrombin complexes (markers of coagulation activation) than HD,^50^ which may be directly caused by local hemodilution,^51^ cellular adhesion to the dialysis membrane, or shear stress.^52^

Our study has several strengths. This study is notable for its use of a natural experiment design where patient characteristics had no effect on the choice of modality between HDF and HD, thereby alleviating the concern for indication bias. We used a statistical analysis that used HDF exposure as a time-varying covariate. Finally, we gathered information over a large sample of individual IRRT sessions which enabled us to detect clinically significant differences in intradialytic events such as intradialytic hypotension.

Our study has also limitations. First, the sample size was relatively limited. Consequently, the possibility remains that HDF may have a clinically significant effect on the risk of mortality or the chances of kidney recovery. This study was of retrospective design and follow-up was incomplete for some patients due to hospital transfer. As discussed previously, HDF was not optimized to achieve the highest convective clearance possible, and it remains unknown if suboptimal HDF may have biased the results toward the null. Some patients had both HD and HDF during the study. Although we accounted for cross-over in our analysis, an inconsistent exposure to HDF could also have biased the results by attenuating the potential benefits of this clearance mode. Finally, we did not investigate whether HDF may have led to increased rates of suboptimal antibiotic dosage leading to possible worse outcomes in septic patients.

The use of HDF was not associated with mortality, kidney recovery, or intradialytic hypotension but was associated with more frequent filter thrombosis. Although a lower subsequent vasopressor use and improved inflammatory status were observed, further investigation is needed to confirm these findings and their effect on meaningful patient outcomes.

## Disclosure

WB-S has received speaker honoraria from Baxter in 2021. RW received unrestricted grant support and speaker fees from Baxter. All the other authors declared no competing interests.

## Supplementary Material

Supplementary File (PDF)

**Table S1.** Characteristics of intermittent renal replacement therapy sessions performed in the ICU in relationship with clearance method

**Table S2.** Association between corrected convection volume as a time-varying exposure and mortality after 90 days

**Table S3.** Association between corrected convection volume as a time-varying exposure and kidney recovery after 90 days

**Table S4.** Association between dilution method and intradialytic hypotension in the ICU

**Table S5.** Association between corrected convection volume and intradialytic hypotension in the ICU

**Table S6.** Association between intermittent renal replacement therapy modality and subsequent vasopressor dependence in the ICU

**Table S7.** Association between intermittent renal replacement therapy modality and subsequent vasopressor dependence in the ICU
