# Supplementary material for: Online Hemodiafiltration Compared to Conventional Hemodialysis in Critically Ill Patients
Source: Kidney Int Rep. 2022 Aug 24;7(11):2376–87. doi: 10.1016/j.ekir.2022.08.007 (PMC9751768; doi:10.1016/j.ekir.2022.08.007)
Supplement: Supplementary File (PDF) [file mmc1.pdf]

Online hemodiafiltration compared to conventional hemodialysis in critically ill patients: A natural experiment – Supplementary material

Table of content

Table S1: Characteristics of intermittent renal replacement therapy sessions performed in the ICU in relationship with clearance method 2

Table S2: Association between corrected convection volume as a time-varying exposure and mortality after 90 days 2

Table S3: Association between corrected convection volume as a time-varying exposure and kidney recovery after 90 days 3

Table S4: Association between dilution method and intra-dialytic hypotension in the ICU 3

Table S5: Association between corrected convection volume and Intra-dialytic hypotension in the ICU ... 4

Table S6: Association between intermittent renal replacement therapy modality and subsequent vasopressor dependence in the ICU 4

Table S7: Association between intermittent renal replacement therapy modality and subsequent vasopressor dependence in the ICU 5

Appendix 1: STROBE checklist 6

Table S1: Characteristics of intermittent renal replacement therapy sessions performed in the ICU in relationship with clearance method

Variable	Hemodialysis	Hemodiafiltration	p-value
**Dalteparin use**	124/316 (39.2%)	195/531 (36.7%)	0.47
**Session duration**	240 (190; 246)	242 (218; 247)	0.08
**Corrected convection volume**	2.1 (0.9; 3.1)	22.7 (17.7; 27.5)	<0.001
**Mean blood flow rate**	350 (300; 400)	350 (310; 400)	0.50

Abbreviations: ICU : intensive care unit

Table S2: Association between corrected convection volume as a time-varying exposure and mortality after 90 days

Variable	Univariable (HR, (95%CI), p)	Multivariable (aHR, (95%CI), p)
**Corrected convection volume (per 5L)**	0.982 (0.957; 1.008) p=0.177	0.893 (0.776; 1.027) p=0.112
**Age**	1.025 (1.000-1.050) p=0.050	1.037 (0.997; 1.077) p=0.067
**Female sex**	1.188 (0.670; 2.106) p=0.555	2.496 (1.173; 5.312) p=0.018
**Charlson comorbitiy score**	1.125 (0.999-1.267) p=0.052	1.126 (0.945; 1.342) p=0.185
**Baseline eGFR**	0.999 (0.989; 1.008) p=0.759	1.013 (1.002; 1.025) p=0.026
**Initially started on CRRT**	0.828 (0.498; 1.378) p=0.468	0.497 (0.240; 1.028) p=0.059
**SOFA score at IRRT initiation**	1.077 (1.000-1.159) p=0.051	1.143 (1.041; 1.255) p=0.005
**Admission diagnostic**		
- **Surgical**	Ref	Ref
- **Medical**	1.620 (0.751; 3.492) p=0.218	2.555 (1.070; 6.102) p=0.035
**Context**		
- **Sepsis**	1.396 (0.796; 2.448) p=0.245	1.180 (0.572; 2/437) p=0.654
- **Heart failure**	0.578 (0.253; 1.319) p=0.193	0.337 (0.096; 1.188) p=0.091
- **Both**	0.697 (0.212; 2.288) p=0.551	0.260 (0.033; 2.053) p=0.201

Cox regression model for count processes with correction convection volume exposure as a time-varying covariate. One patient was excluded from the analysis for loss to follow-up after a singular IRRT session (N=181).

Abbreviations: eGFR : estimated glomerular filtration rate; CRRT: continuous renal replacement therapy; SOFA : sequential organ failure assessment score; IRRT : intermittent renal replacement therapy; HR : hazard ratio ; aHR : adjusted hazard ratio; CI : confidence interval

Table S3: Association between corrected convection volume as a time-varying exposure and kidney recovery after 90 days

Variable	Univariable (HR, (95%CI), p)	Multivariable (aHR, (95%CI), p)
**Corrected convection volume (per 5L)**	1.004 (0.987; 1.021) p=0.644	1.017 (0.935; 1.107) p=0.694
**Age**	0.986 (0.973; 0.999) p=0.033	0.993 (0.972; 1.014) p=0.498
**Female sex**	1.068 (0.709; 1.607) p=0.754	1.336 (0.842; 2.121) p=0.219
**Charlson comorbitiy score**	0.945 (0.874; 1.022) p=0.159	1.030 (0.918; 1.156) p=0.616
**Baseline eGFR**	1.008 (1.002; 1.014) p-0.007	1.008 (1.001; 1.015) p=0.023
**Initially started on CRRT**	1.002 (0.826; 1.216) p=0.984	0.646 (0.417; 1.000) p=0.050
**SOFA score at IRRT initiation**	1.010 (0.959; 1.064) p=0.696	1.007 (0.950; 1.068) p=0.810
**Admission diagnostic**		
- **Surgical**	Ref	Ref
- **Medical**	1.063 (0.859; 1.315) p=0.573	1.279 (0.789; 2.075) p=0.318
**Context at ICU admission**		
- **Sepsis**	1.227 (0.780; 1.931) p=0.376	1.372 (0.847; 2.24) p=0.199
- **Heart failure**	0.929 (0.556; 1.552) p=0.778	1.023 (0.592; 1.767) p=0.936
- **Both**	1.694 (0.829; 3.461) p=0.148	1.981 (0.936; 4.192) p=0.074

Cox regression model for count processes with correction convection volume exposure as a time-varying covariate. Two patients were excluded from the analysis for kidney recovery and loss to follow up immediately after the first IRRT session (N=180).

Abbreviations: eGFR : estimated glomerular filtration rate; CRRT: continuous renal replacement therapy; SOFA : sequential organ failure assessment score; IRRT : intermittent renal replacement therapy; HR : hazard ratio ; aHR : adjusted hazard ratio; CI : confidence interval

Table S4: Association between dilution method and intra-dialytic hypotension in the ICU

	Univariable (OR, (95%CI), p)	Multivariable (aOR, (95%CI), p)
**Standard hemodialysis**	Ref	Ref
**Pre-dilution HDF**	0.872 (0.581-1.309) p=0.509	0.847 (0.560-1.280) p=0.429
**Post-dilution HDF**	1.007 (0.665-1.525) p=0.975	0.979 (0.656-1.462) p=0.918
**Mechanical ventilation**	1.377 (0.914-2.076) p=0.126	1.278 (0.853-1.915) 0.235
**Vasopressor-inotropic score (per 1 point increase)**	1.068 (1.003-1.138) p=0.041	1.061 (0.997-1.129) p=0.064
**Duration since IRRT initiation (days)**	0.990 (0.973-1.007) p=0.247	0.991 (0.975-1.008) p=0.303

Data from 848 IRRT sessions in 182 patients was included.

Abbreviations: ICU: intensive care unit; HDF: hemodiafiltration; IRRT : intermittent renal replacement therapy; OR : odds ratio ; aOR : adjusted odds ratio; CI : confidence interval

Table S5: Association between corrected convection volume and Intra-dialytic hypotension in the ICU

	Univariable (OR, (95%CI), p)	Multivariable (aOR, (95%CI), p)
**Corrected convection volume (L/session)**	0.992 (0.978-1.006) p=0.256	0.992 (0.979-1.001) p=0.246
**Mechanical ventilation**	1.377 (0.914-2.076) p=0.126	1.278 (0.853-1.915) 0.235
**Vasopressor-inotropic score (per 1 point increase)**	1.068 (1.003-1.138) p=0.041	1.061 (0.997-1.129) p=0.064
**Duration since IRRT initiation (days)**	0.990 (0.973-1.007) p=0.247	0.991 (0.975-1.008) p=0.303

Data from 848 IRRT sessions in 182 patients was included.

Abbreviations: ICU : intensive care unit; IRRT : intermittent renal replacement therapy; OR : odds ratio ; aOR : adjusted odds ratio; CI : confidence interval

Table S6: Association between intermittent renal replacement therapy modality and subsequent vasopressor dependence in the ICU

	Univariable (OR, (95%CI), p)	Multivariable (aOR, (95%CI), p)
**Standard hemodialysis**	Ref	Ref
**Pre-dilution HDF**	0.644 (0.377-1.099) p=0.107	0,646 (0,375-1,114) p=0.116
**Post-dilution HDF**	0.562 (0.302-1.048) p=0.070	0,551 (0,289-1,051) p=0.070
**Vasopressor-inotropic score (per 1 point increase)**	1.030 (0.991-1.070) p=0.130	1,028 (0,994-1,064) p=0.110
**Duration since IRRT initiation (days)**	0.976 (0.896-1.064) p=0.588	0,968 (0,892-1,051) p=0.439

Data from 848 IRRT sessions in 182 patients was included.

Abbreviations: HDF: hemodiafiltration; IRRT : intermittent renal replacement therapy; OR : odds ratio ; aOR : adjusted odds ratio; CI : confidence interval

Table S7: Association between intermittent renal replacement therapy modality and subsequent vasopressor dependence in the ICU

	**Univariable (OR, (95%CI), p)**	**Multivariable (aOR, (95%CI), p)**
**Corrected convection volume**	0.990 (0.970-1.012) p=0.354	0.990 (0.969-1.012) p=0.376
**Vasopressor-inotropic score (per 1 point increase)**	1.030 (0.991-1.070) p=0.130	1.029 (0.992-1.067) p=0.127
**Duration since IRRT initiation (days)**	0.976 (0.896-1.064) p=0.588	0.974 (0.896-1.058) p=0.531

Data from 848 IRRT sessions in 182 patients was included.

Abbreviations: IRRT : intermittent renal replacement therapy; OR : odds ratio ; aOR : adjusted odds ratio; CI : confidence interval

Appendix 1: STROBE checklist

STROBE Statement—Checklist of items that should be included in reports of ***cohort studies***

	Item No	Recommendation	Page No
**Title and abstract**	1	(*a*) Indicate the study's design with a commonly used term in the title or the abstract<br>(*b*) Provide in the abstract an informative and balanced summary of what was done and what was found	1, 3
**Introduction**			
Background/rationale	2	Explain the scientific background and rationale for the investigation being reported	4
Objectives	3	State specific objectives, including any prespecified hypotheses	4
**Methods**			
Study design	4	Present key elements of study design early in the paper	4
Setting	5	Describe the setting, locations, and relevant dates, including periods of recruitment, exposure, follow-up, and data collection	4-5
Participants	6	(*a*) Give the eligibility criteria, and the sources and methods of selection of participants. Describe methods of follow-up<br>(*b*) For matched studies, give matching criteria and number of exposed and unexposed	4-5
Variables	7	Clearly define all outcomes, exposures, predictors, potential confounders, and effect modifiers. Give diagnostic criteria, if applicable	5-6
Data sources/ measurement	8*	For each variable of interest, give sources of data and details of methods of assessment (measurement). Describe comparability of assessment methods if there is more than one group	5
Bias	9	Describe any efforts to address potential sources of bias	6-7
Study size	10	Explain how the study size was arrived at	5
Quantitative variables	11	Explain how quantitative variables were handled in the analyses. If applicable, describe which groupings were chosen and why	6-7
Statistical methods	12	(*a*) Describe all statistical methods, including those used to control for confounding<br>(*b*) Describe any methods used to examine subgroups and interactions<br>(*c*) Explain how missing data were addressed<br>(*d*) If applicable, explain how loss to follow-up was addressed	6-7

		(*e*) Describe any sensitivity analyses	
**Results**			
Participants	13*	(a) Report numbers of individuals at each stage of study—eg numbers potentially eligible, examined for eligibility, confirmed eligible, included in the study, completing follow-up, and analysed<br>(b) Give reasons for non-participation at each stage<br>(c) Consider use of a flow diagram	7 fig 1
Descriptive data	14*	(a) Give characteristics of study participants (eg demographic, clinical, social) and information on exposures and potential confounders<br>(b) Indicate number of participants with missing data for each variable of interest<br>(c) Summarise follow-up time (eg, average and total amount)	7, Table 1
Outcome data	15*	Report numbers of outcome events or summary measures over time	8-9
Main results	16	(*a*) Give unadjusted estimates and, if applicable, confounder-adjusted estimates and their precision (eg, 95% confidence interval). Make clear which confounders were adjusted for and why they were included<br>(*b*) Report category boundaries when continuous variables were categorized<br>(*c*) If relevant, consider translating estimates of relative risk into absolute risk for a meaningful time period	8-9<br>Table 2-7
Other analyses	17	Report other analyses done—eg analyses of subgroups and interactions, and sensitivity analyses	8-9
**Discussion**			
Key results	18	Summarise key results with reference to study objectives	10-11
Limitations	19	Discuss limitations of the study, taking into account sources of potential bias or imprecision. Discuss both direction and magnitude of any potential bias	11
Interpretation	20	Give a cautious overall interpretation of results considering objectives, limitations, multiplicity of analyses, results from similar studies, and other relevant evidence	10-11
Generalisability	21	Discuss the generalisability (external validity) of the study results	10-11
**Other information**			
Funding	22	Give the source of funding and the role of the funders for the present study and, if applicable, for the original study on which the present article is based	12
